# Improved Murine Model for the Intravital Microscopic Examination of Manifest Tumors

**DOI:** 10.3390/cells14191556

**Published:** 2025-10-08

**Authors:** Frank Tavassol, Jan Winterboer, Philipp Jehn, Matthias Kappler, Felix Tilsen, Andreas Kampmann

**Affiliations:** 1 Department of Oral and Maxillofacial Surgery, Faculty of Medicine, Martin Luther University Halle-Wittenberg, 06120 Halle, Germany; matthias.kappler@uk-halle.de (M.K.); felix.tilsen@uk-halle.de (F.T.); 2 Department of Oral and Maxillofacial Surgery, Hannover Medical School, 30625 Hannover, Germanyjehn.philipp@mh-hannover.de (P.J.); kampmann.andreas@mh-hannover.de (A.K.)

**Keywords:** dorsal skinfold chamber, tumor model, intravital microscopy

## Abstract

Animal models are essential for studying tumor pathophysiology; however, most lack the capacity for repeated in vivo observation of tumor growth and vascularization over extended periods. This study aimed to establish a novel in vivo model using the mouse dorsal skinfold chamber. Tumor induction was performed using different membrane types (two polytetrafluoroethylene meshes and a polydioxanone plate), followed by monitoring of tumor vascularization via intravital fluorescence microscopy (IVM). Tumors developed successfully over six weeks, demonstrating sustained vascular supply and enabling, for the first time, the investigation of vascular networks in advanced tumors. Among the membranes tested, the polydioxanone membrane facilitated easier chamber preparation but may negatively affect angiogenesis and promote inflammation. IVM revealed persistent microcirculation in manifested tumors over six consecutive days, allowing detailed assessment of microvascular parameters, leukocyte–endothelial interactions, and functional capillary density. This model enables repetitive, high-resolution visualization of tumor microcirculation dynamics in vivo. In conclusion, this improved mouse dorsal skinfold chamber combined with IVM provides a powerful tool for investigating tumor angiogenesis and evaluating therapeutic interventions in advanced tumors.

## 1. Introduction

Head and neck malignancies are the fourth most common type of cancer, accounting for 4.8% of all cancers worldwide [1]. The annual incidence exceeds 550,000 cases, resulting in approximately 300,000 deaths each year. Roughly 90% of head and neck malignancies are squamous cell carcinomas [1,2]. Despite advances in therapy in recent years, the 5-year survival rate remains unchanged at around 50%. Surgery is still the treatment of choice in most clinical cases, but it offers limited benefit in advanced tumors. Due to the anatomical complexity of the head and neck region, surgical interventions often lead to significant functional impairments, while both chemotherapy and radiotherapy demonstrate limited efficacy.

Exogenous risk factors such as tobacco smoking and chronic alcohol consumption are the main contributors to the development of oral squamous cell carcinoma (OSCC) [3]. Additional risk factors include exposure to dust, vapors, ionizing radiation, poor oral hygiene, and infection with human papillomavirus or Epstein–Barr virus [4].

Tumor-induced angiogenesis is essential for tumor growth and metastasis. Angiogenesis refers to the sprouting of capillaries from pre-existing vessels, followed by the recruitment of stabilizing cells (pericytes and smooth muscle cells). This neovascularization process leads to the formation of a tubular vessel lumen, the onset of blood flow, and the establishment of a basement membrane. Once the pro-angiogenic stimulus subsides, angiogenesis ceases [5,6]. Under physiological conditions, angiogenesis is tightly regulated and self-limiting due to a precise balance between pro- and anti-angiogenic factors. However, under pathological conditions such as chronic inflammation, impaired wound healing, or tumor growth, angiogenesis becomes persistently upregulated [7,8].

The dorsal skinfold chamber has been widely used to study tumor growth and angiogenesis in vivo by implanting tumor cells. Typically, moderate tumor growth can be observed after 14 days [9,10,11,12]. The combination of the dorsal skinfold chamber and intravital fluorescence microscopy (IVM) enables real-time visualization of dynamic cellular behaviors and vascularization in living tissue using multiple fluorescence markers, laser-based light sources, and advanced detection systems [13,14]. These methods have become key tools in studying tumor-induced angiogenesis, metastasis, and evaluating anti-angiogenic therapies in murine models [10,15,16]. Similar models include the cranial window and femoral chamber, which also allow the study of microcirculation in rodents [17,18].

However, dorsal skinfold chamber models are limited to a maximum observation period of approximately three weeks due to skin tension loss, infection risk, edema formation, and neovascularization at the chamber rim [10]. Consequently, these models have primarily been used to analyze angiogenesis induced by implanted tumor cells or tissue fragments, covering only short-term development—not the progression of advanced tumors with mature vascular networks. This limitation restricts the relevance of the model for investigating anti-angiogenic therapies, which are clinically applied to fully developed tumors with complex micro- and macrocirculation.

Given these limitations, there is a critical need for animal models that enable repetitive, long-term intravital observation of tumor microcirculation under therapeutic conditions. Such models would provide valuable insights into the complex interactions between tumor cells, endothelial cells, and an established vascular network, and are essential for the development of effective anti-angiogenic therapies [19].

This study presents the first model allowing repetitive IVM-based visualization of physiological microcirculation in advanced tumors in situ, providing a valuable tool for the preclinical evaluation of anti-angiogenic therapies.

## 2. Materials and Methods

### 2.1. Cell Cultures and Carrier

**Figure 1:**
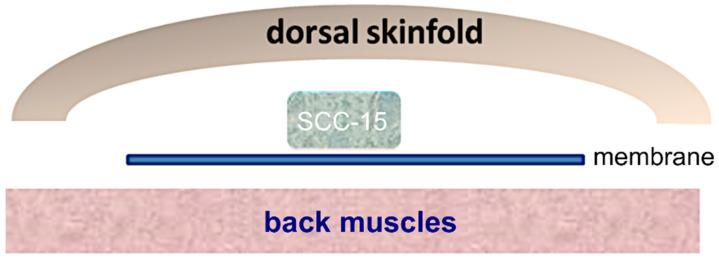
Schematic representation of the implanted tumor cells (SCC-15: human cells of a squamous cell carcinoma of the tongue, applied on a gelatin carrier).

The SCC-15 cell line (CRL-1623™) was isolated from a squamous cell carcinoma of the tongue [20] and obtained commercially from the American Type Culture Collection (ATCC, Rockville, MD, USA). A resorbable, hemostatic gelatin sponge (Spongostan™; Ferrosan Medical Devices A/S, Soeborg, Denmark) was used as carrier material for the cell line. Spongostan™ is a medical device approved for human and animal use. Briefly, 1 × 10^6^ cells in Matrigel (10 µL; Corning GmbH, Kaiserslautern, Germany) were applied on the carrier (size approx. 3 mm × 3 mm × 2 mm), which was then attached to a surgical membrane (size approx. 5 mm × 5 mm) and implanted under the dorsal skin of the mice (Figure 1).

Successful tumor induction and subsequent preparation of the skinfold chamber depended on the direction of growth of the induced tumor towards the dorsal skin. To this end, we tested different surgical membranes, such as those made of expanded polytetrafluoroethylene (GORE^®^ Dualmesh^®^, GORE^®^ Preclude^®^; W. L. Gore & Associates, Inc., Flagstaff, AZ, USA), and polydioxanone (PDS^®^ plate; Johnson & Johnson Medical GmbH, Norderstedt, Germany). Membranes that prevented tumor adhesion to the membrane surface and to the underlying muscular layers at the back of the animals were selected. This ensured that the tumor grew in a targeted manner attached to the dorsal skin and was still connected to the blood supply after preparation of the skinfold chamber. Furthermore, as they are slowly absorbable, these membranes retained their function over the entire period of tumor growth, before chamber preparation. To determine which of the membranes was most suitable for this project, three groups with five animals each were formed. As this study was designed as a pilot study, statistical planning of group sizes was not possible due to the lack of comparative data. The calculation of the number of animals was based on the extensive experience of our research group with the dorsal skinfold chamber model.

### 2.2. Animals

All experiments were conducted in accordance with the German legislation for the protection of animals and the Guide for the Care and Use of Laboratory Animals (8th edition, 2011). The experiments were approved by the competent authority (Niedersächsisches Landesamt für Verbraucherschutz und Lebensmittelsicherheit (Lower Saxony State Office for Consumer Protection and Food Safety), reference number 33.12-42502-04-16/2276) and were carried out in accordance with ARRIVE guidelines. A total of 12 NSG mice (NOD.Cg-Prkdc^scid^ Il2rg^tm1WjI^) (Zentrales Tierlabor, Hannover Medical School) of mixed sex, aged at least 12 weeks at the beginning of the experiment and with a body weight of 30–35 g were used for the study. The mice were randomly allocated to experimental groups; no animal had to be excluded from the study. The animals were kept in a conditioned environment (room temperature: 25.8 °C, relative humidity: 50–70%), under a 12 h light–dark cycle, and in single cages (Macrolon cages Type II; Techniplast, Buguggiate, Italy). Throughout the experimental period, the animals had access to standardized dry food (1328 Hybridpellet; Altromin, Lage, Germany) and water ad libitum.

### 2.3. Anesthesia

Inhalation of isoflurane was used in all procedures and manipulations that required anesthesia. Briefly, the animals were placed in an induction box containing 4–5% isoflurane in oxygen and left there until fully anesthetized. Animals remained anesthetized with the aid of a nose cone with 2–3% isoflurane in oxygen. To prevent the conjunctiva from drying out, an ointment (Bepanthen^®^, Bayer, Leverkusen, Germany) was applied when necessary.

### 2.4. Tumor Induction, Dorsal Skinfold Chamber

After the animals had been anesthetized, the back was shaved, depilated, and disinfected. In the prone position, the dorsal skinfold of the test animals was raised under backlight control, and the location of the future dorsal skinfold chamber observation window was marked. A subcutaneous pocket was prepared in the thoracolumbar region via an approximately 5 mm long incision in the back area, into which the prepared cell carrier was implanted, aligned, and fixed with two stitches of non-resorbable sutures (Prolene^®^ monofilblau 6-0; Ethicon, Johnson & Johnson, Bridgewater, NJ, USA). The wound was then sutured. Perioperative management was carried out taking animal welfare into account.

**Figure 2:**
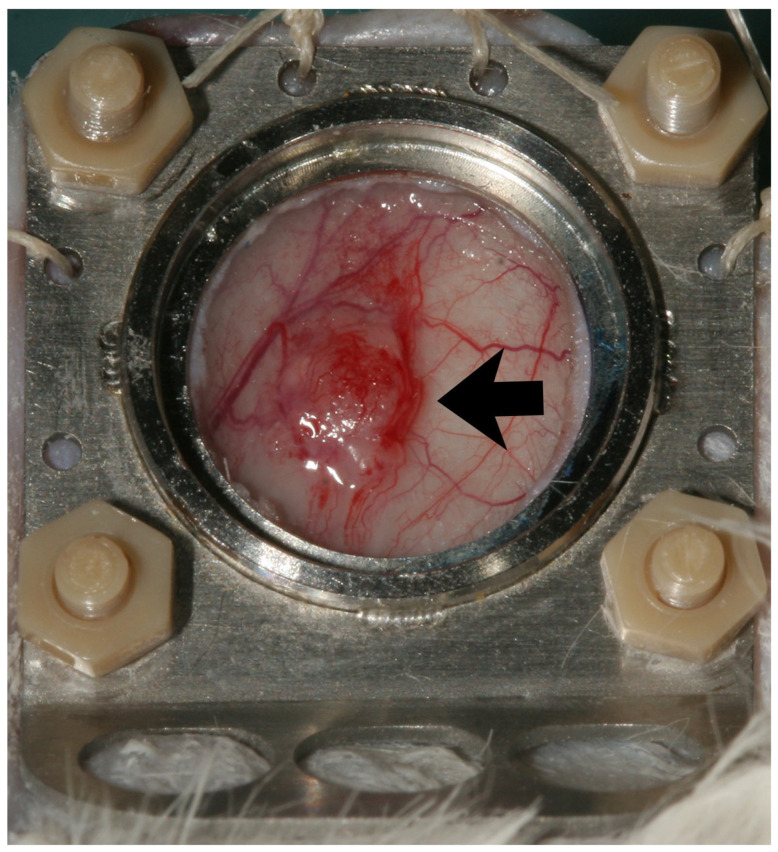
Observation window with macroscopic visible tumor (arrow) on day 0. Diameter of the observation window is 12 mm.

The animals were recovered under an infrared heat lamp within a few minutes after inhalation anesthesia was discontinued. Shortly thereafter, the animals showed inconspicuous eating and drinking, as well as appropriate cleaning and exploration, behavior. In the following 4–6 weeks, a clearly visible and palpable manifest tumor grew from the implanted cells (Figure 2); its size was measured twice a week.

Following this initial 6-week period, the dorsal skinfold chamber was implanted as described before [20]. During the preparation, particular care was taken to ensure that the macroscopically visible tumor was located exactly in the center of the observation window. The intravital microscopy protocol using FITC-dextrane and Rhodamin-6G was performed as described before [20]. However, as this was a pilot study, the trial only lasted 6 days and not 14 as done previously.

### 2.5. Intravital Fluorescence Microscopy

Intravital fluorescence microscopy was carried out on day 0, 3, and 6. Microhemodynamics, inflammatory parameters (leukocyte–endothelial cell interactions), and functional capillary density were recorded as described previously [20].

Only vessels with a diameter of 20–50 µm were selected. Data were analyzed using CapImage 8.6.3 software (Zeintl, Heidelberg, Germany).

### 2.6. Analysis of Microcirculatory Parameters

The recorded data were analyzed by means of the image analysis software CapImage (CapImage 8.6.3., Zeintl, Heidelberg Germany). Leukocyte–endothelial cell interactions, microhemodynamics, and macromolecular leakage were measured in four different regions of interest in the periphery of the tumor by using 2.5×, 10×, and 20× objectives. In each region of interest, one venule (inner diameter: 20–40 µm) was selected and observed over 20 s for evaluation of vessel diameter, red blood cell velocity, wall shear rate, and macromolecular leakage. The leukocytes were classified according to their interaction with the vascular endothelium as adherent, rolling, or free flowing cells. Adherent leukocytes were cells that did not move or detach from the endothelial lining within an observation period of 20 s. Rolling leukocytes were moving cells with a velocity less than two-fifths of the centerline velocity. Diameters (d) were analyzed in micrometers perpendicular to the vessel path. The velocity (v) was measured using the line-shift method [21]. Volumetric blood flow was calculated by $Q=\pi \times {\left(d/2\right)}^{2}\times \frac{v}{1.6}[\frac{pl}{s}]$ For correction of the parabolic velocity profile in microvessels, the Baker–Wayland factor 1.6 was used [22]. Based on the Newtonian definition, the wall shear rate was calculated by $\;y=8\times \frac{v}{d}$. Macromolecular leakage was analyzed by means of grey levels in the tissue directly adjacent to the venular vessel wall (E_1_) and in the marginal cell-free plasma layer within the blood vessel (E_2_). Extravasation (E) was calculated as E = E_1_/E_2_. Microvessel density, defined as the length of blood vessels per area of observation given in cm/cm^2^, was measured in the periphery around the implant and in the center of the implant. Both values were displayed as the total functional capillary density expressed as the sum of peripheral and central functional capillary density.

### 2.7. Histology

At the end of the experimental period, animals were euthanized via cervical dislocation under deep anesthesia and histological examinations were performed. Formalin-fixed specimens of the dorsal skinfold chamber were embedded in paraffin according to standard procedures. Thin sections (5 µm) were stained with hematoxylin and eosin (Merck KGaA, Darmstadt, Germany) following standard protocols and examined by light microscopy (Leica DM4000 B; Leica Microsystems, Wetzlar, Germany).

Neoplastic cells were detected using a mouse anti-human p53 antibody with a dilution of 1:200 (Dianova GmbH, Hamburg, Germany). A secondary biotin-conjugated goat anti-mouse antibody (dilution 1:200) was used (Dianova GmbH, Hamburg, Germany). After incubation with streptavidin-conjugated horseradish peroxidase (Dianova GmbH, Hamburg, Germany), color development with the addition of 3.3′-diaminobenzidine (Vector Laboratories, Inc., Burlingame, CA, USA) was monitored microscopically, followed by counterstaining with hematoxylin.

A rabbit anti-human pan-cytokeratin antibody with a dilution of 1:100 (Acris Antibodies GmbH, Herford, Germany) was used to detect SCC-15 cells. For immunofluorescence detection, an Alexa Fluor^®^ 488-conjugated goat anti-rabbit antibody (dilution 1:200, Dianova GmbH, Hamburg, Germany) was used. Nuclei were stained using DAPI (Carl Roth GmbH, Karlsruhe, Germany).

Capillaries were visualized using a rabbit anti-mouse CD31 antibody with a dilution of 1:50 (LifeSpan Biosciences, purchased from BIOZOL Diagnostica Vertrieb GmbH, Eching, Germany). A secondary biotin-conjugated goat anti-rabbit antibody (dilution 1:200) was used (Dianova GmbH, Hamburg, Germany). After incubation with streptavidin-conjugated horseradish peroxidase (Dianova GmbH, Hamburg, Germany), color development with the addition of 3.3′-diaminobenzidine (Vector Laboratories, Inc., Burlingame, CA, USA) was monitored microscopically followed by counterstaining with hematoxylin.

Negative controls were performed by omitting the primary antibody, and they all showed no detectable staining. All specimens were examined by light or fluorescence microscopy, respectively (Leica DM4000 B; Leica Microsystems, Wetzlar, Germany).

### 2.8. Statistical Analysis

Results are expressed as means ± SEM, unless otherwise stated. Differences between groups were assessed by one-way ANOVA, whereas differences within each group were analyzed by one-way repeated-measures ANOVA. Student–Newman–Keuls or Dunn’s post hoc tests were used to point to specific differences. Results with *p* < 0.05 were considered significant.

## 3. Results

### 3.1. Clinical Findings

Within the first six weeks after tumor cell implantation, all animals developed macroscopically visible tumors using three different cell carrier materials (1. GORE^®^ Dualmesh^®^ or 2. GORE^®^ Preclude^®^) and polydioxanone (3. PDS^®^ plate) (Figure 2).

In one mouse, tumor cell carry-over occurred during tumor induction, resulting in a second tumor in the area of the incision site. However, this could be excised during the preparation of the dorsal skinfold chamber, allowing continued visualization of the primary tumor.

In all three groups using different cell carrier materials, sufficient mobility between the respective membrane and the underlying muscle layers of the animal’s back allowed for the formation of a skinfold and, consequently, the implantation of the dorsal skinfold chamber. However, there were significant differences in handling of the three membranes.

Ideally, the membrane should detach from the tumor as gently as possible to avoid hindering blood supply to the tumor. PDS^®^ plate proved to be most suitable, because it could be removed easily from the tumor, while the Dualmesh^®^ membrane had a pronounced adherence to the tumor surface. The Preclude^®^ membrane frequently developed wrinkles, which complicated the preparation procedure. In two of the four animals examined in this group, small remnants of the membrane had to be left in place and could not be removed in order to avoid damaging the tumor tissue and the supplying vessels. Following the end of inhalation anesthesia, some animals showed delayed recovery, but all recovered shortly thereafter. The dorsal skinfold chamber was well-tolerated by all animals. In three of the 12 animals, mild irritations occurred in the area of the dorsal skin or wound margins, which presented as slight swelling or redness. However, these had no impact on the animals’ well-being or wound healing and resolved within two days. Vascular supply remained intact, and the animal did not need to be excluded from the study.

### 3.2. Microhemodynamics

Over the 6-day observation period, venule diameters showed no significant differences among the three investigated membrane groups: polytetrafluoroethylene-based (1) GORE^®^ Dualmesh^®^, (2) GORE^®^ Preclude^®^, and (3) polydioxanone (PDS^®^ plate). The velocity of blood cells ranged from 300 to 600 µm/s, with no significant differences observed among the groups (Table 1).

However, the velocity of blood cells increased over time of the experiment (6 days), rising from 354 ± 68 µm/s on day 0 to 491 ± 112 µm/s on day 6 across all three groups (mean value). The post-capillary wall shear rate ranged between 50 and 150 s^−1^. Significant differences were observed in volumetric blood flow (120–300 pL/s) (Table 2). It was found that the application of PDS^®^ plate resulted in a significantly lower blood flow compared to both polytetrafluoroethylene membranes groups on day 6 of the experiment (Table 2).

For both polytetrafluoroethylene membranes, the blood flow increased over time from 128 ± 56 pL/s (day 0) to 290 ± 99 pL/s on day 6. In contrast, the blood flow remained almost unchanged in the PDS^®^ plate group over the period of six days (Table 2).

### 3.3. Inflammatory Response

**Figure 3:**
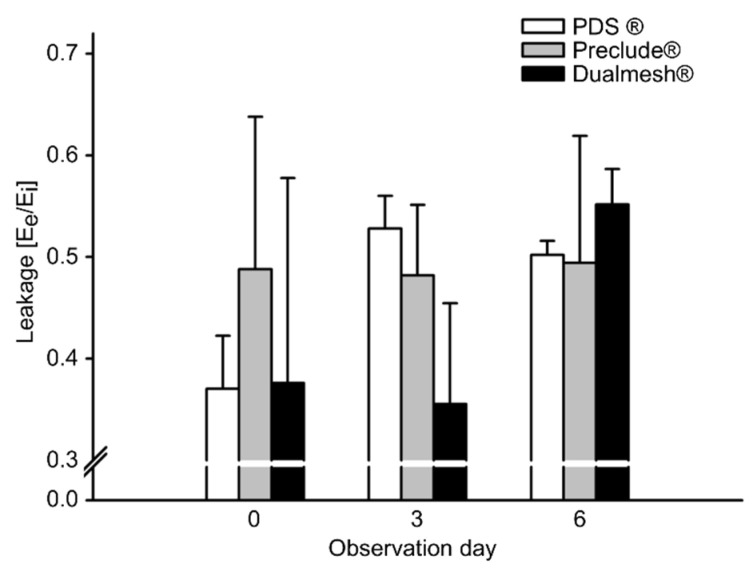
Macromolecular leakage as an indicator of microvascular permeability in post-capillary and collecting venules at the periphery of the tumor. Values represent means ± SEM.

Throughout the experiment, no significant changes in vascular permeability were detected among the three groups. There was a slight, but not significant increase in permeability in the PDS^®^ plate group (day 0 to day 3) and in the Dualmesh^®^ membrane group (day 3 to day 6, Figure 3).

**Figure 4:**
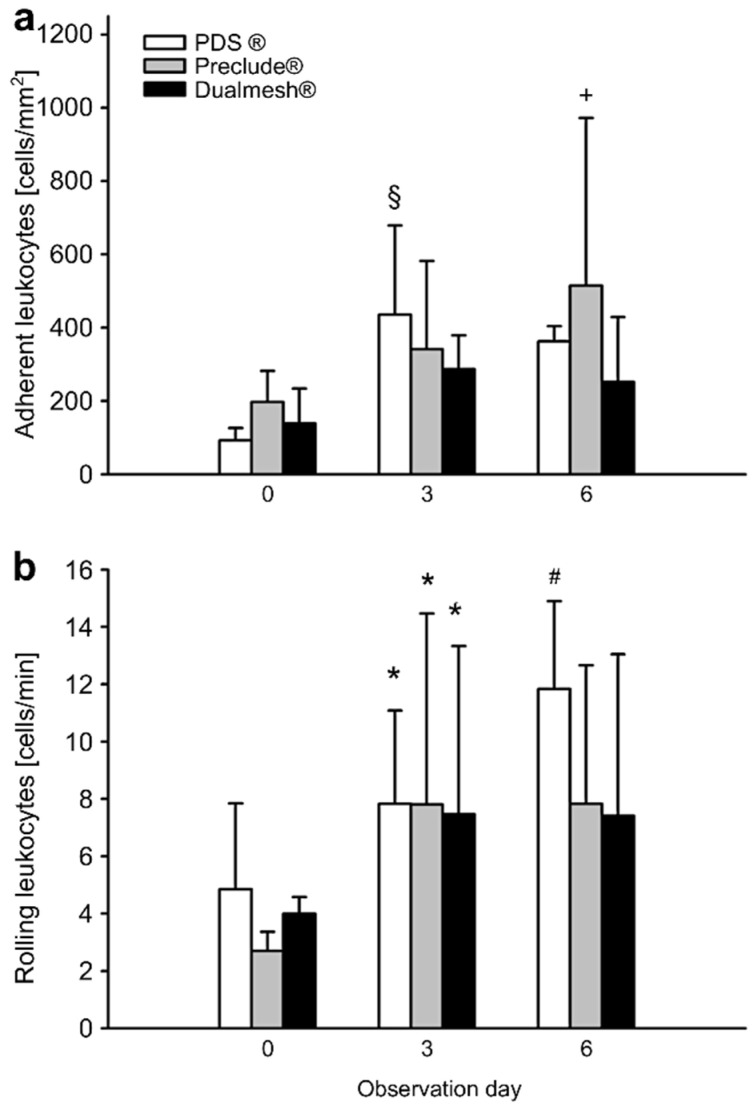
The number of adherent and rolling leukocytes. (**a**) Average number of adherent leukocytes [cells/mm^2^] in post-capillary venules and collecting venules over the observation period of 6 days. Values represent means ± SD. ^§^ *p* < 0.05 vs. Preclude^®^, PDS^®^, and Dualmesh^®^ on day 0 and Dualmesh^®^ on day 3 and day 6. ^+^ *p* < 0.05 vs. Preclude^®^, PDS^®^, and Dualmesh^®^ on day 0. (**b**) Average number of rolling leukocytes [cells/min] in post-capillary venules and collecting venules over the observation period of 6 days. Values represent means ± SD. * *p* < 0.05 vs. Preclude^®^, PDS^®^, and Dualmesh^®^ on day 0 and day 3. ^#^ *p* < 0.05 vs. Preclude^®^ and Dualmesh^®^ on day 0.

In addition, the number of rolling leukocytes, as opposed to those adhering to the endothelium of post-capillary venules, was analyzed. In the Preclude^®^ group, the number of adherent leukocytes increased significantly over the observation period. In contrast, the PDS^®^ and Dualmesh^®^ groups showed a decrease in adherent leukocytes by day 6 compared to day 3 of the experiment (Figure 4a). Rolling leukocytes showed an overall increase over the observation period, with a significant rise on day 3 in all groups compared to day 0 (Figure 4b).

### 3.4. Angiogenesis

**Figure 5:**
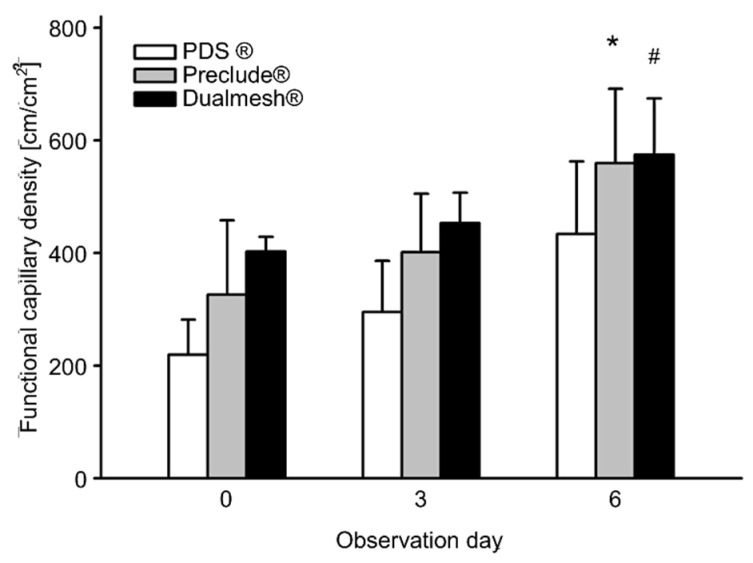
Functional capillary density [cm/cm^2^] over the observation period of 6 days. Values represent means ± SD. * *p* < 0.05 vs. Preclude^®^ and PDS^®^ on day 0 and PDS^®^ on day 3. ^#^ *p* < 0.05 vs. Preclude^®^ and PDS^®^ on day 0 and PDS^®^ on day 3.

**Figure 6:**
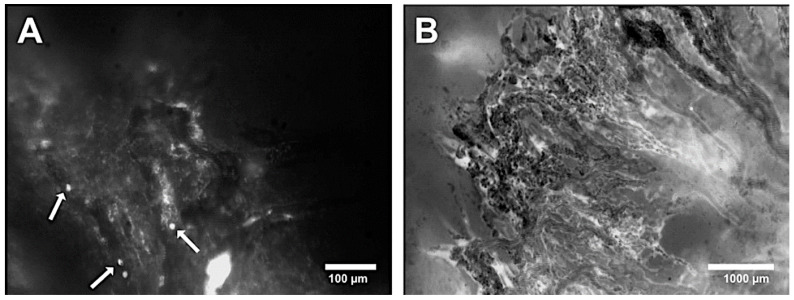
Intravital fluorescence microscopy of vessels in a tumor from the PDS^®^ group on day 3. (**A**) Leucocyte recruitment appeared as bright spots within the vessel lumen (arrows). (**B**) Overview of the manifest tumor showing strong vascular supply.

Functional capillary density was used to quantify the angiogenetic effect of the tumor. At the beginning of intravital microscopy, pronounced vascularization was already evident due to previous tumor growth. The measurement ranged from 200 to 400 cm/cm^2^, with both polytetrafluoroethylene membranes showing higher capillary density compared to the PDS membrane; even on day 0 of the experiment. Over time, all groups show an increase in functional capillary density, indicating a progressive tumor vascularization. By day 6, both Preclude^®^ and Dualmesh^®^ (both polytetrafluoroethylene membranes) groups showed significant increases in capillary density compared to the previous time points (Figure 5 and Figure 6).

### 3.5. Histology and Immunohistology

**Figure 7:**
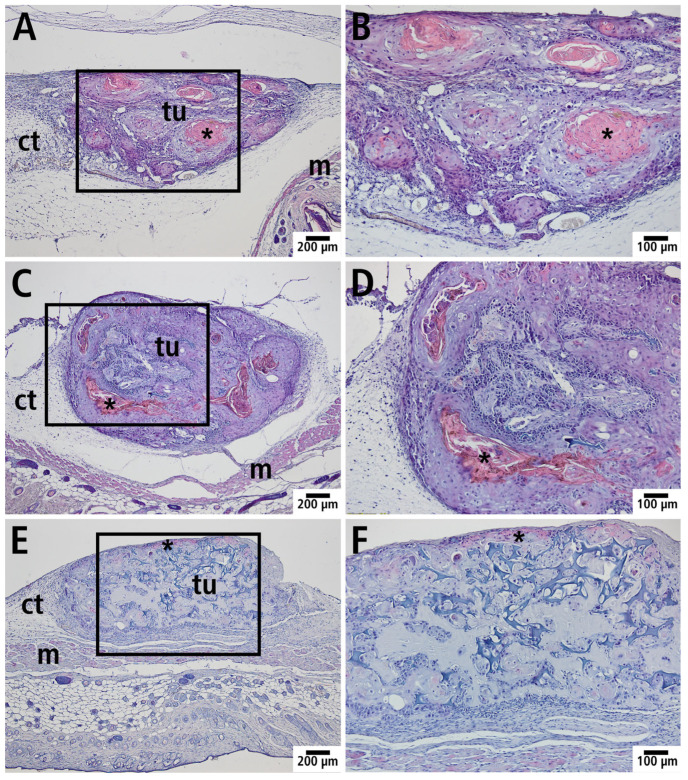
Histological section (hematoxylin and eosin staining) with manifest tumor (tu) in the PDS^®^ group (**A**,**B**), Preclude^®^ group (**C**,**D**), and Dualmesh^®^ group (**E**,**F**); m: striated muscle, ct: connective tissue. (**B**,**D**,**F**): Magnification of the marked area (black rectangle) of the histological sections from (**A**,**C**,**E**). * Horn slat of keratinizing squamous cell carcinoma.

After completing intravital fluorescence microscopy on day 6, the tumors and their surrounding tissue were prepared for histological studies. In hematoxylin and eosin-stained sections, squamous cell carcinoma formations in connective tissue, together with associated structures, such as striated muscles and connective tissue in close vicinity to the tumor, were clearly visible (Figure 7).

**Figure 8:**
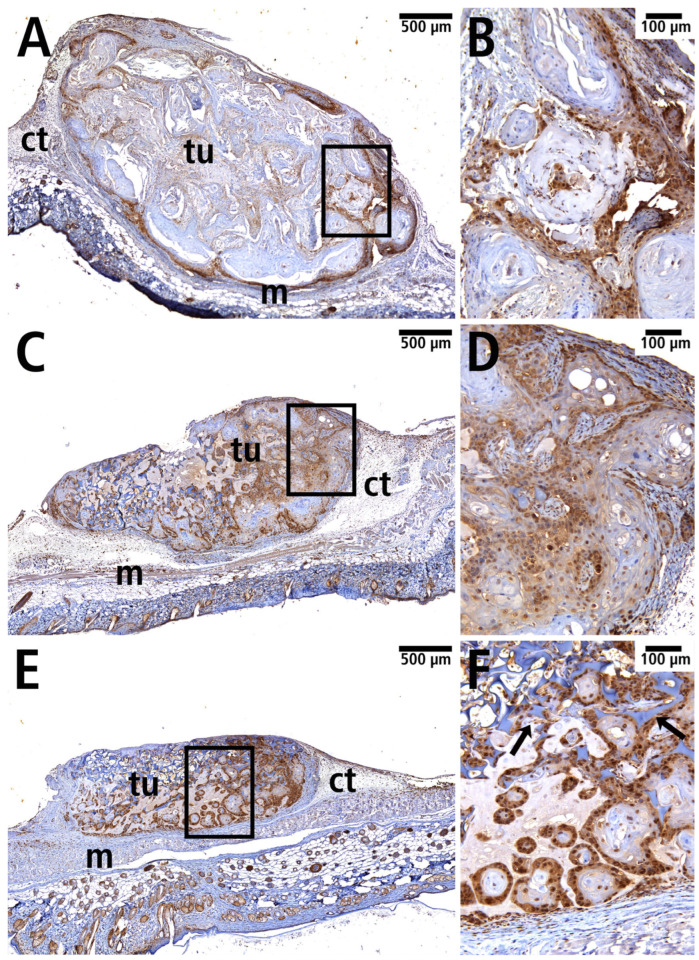
Immunohistochemically labeled p53 protein within the manifest tumor (tu) in the PDS^®^ group (**A**,**B**), Preclude^®^ group (**C**,**D**), and Dualmesh^®^ group (**E**,**F**); m: striated muscles, ct: connective tissue. (**B**,**D**,**F**): Magnification of the marked area (black rectangle) of the histological sections from (**A**,**C**,**E**). Remnants of Spongostan™ are highlighted ((**F**), arrows).

To visualize neoplastic cells, tissue sections were immunostained for the tumor suppressor protein p53. Expression of p53 was detected in all samples (Figure 8).

**Figure 9:**
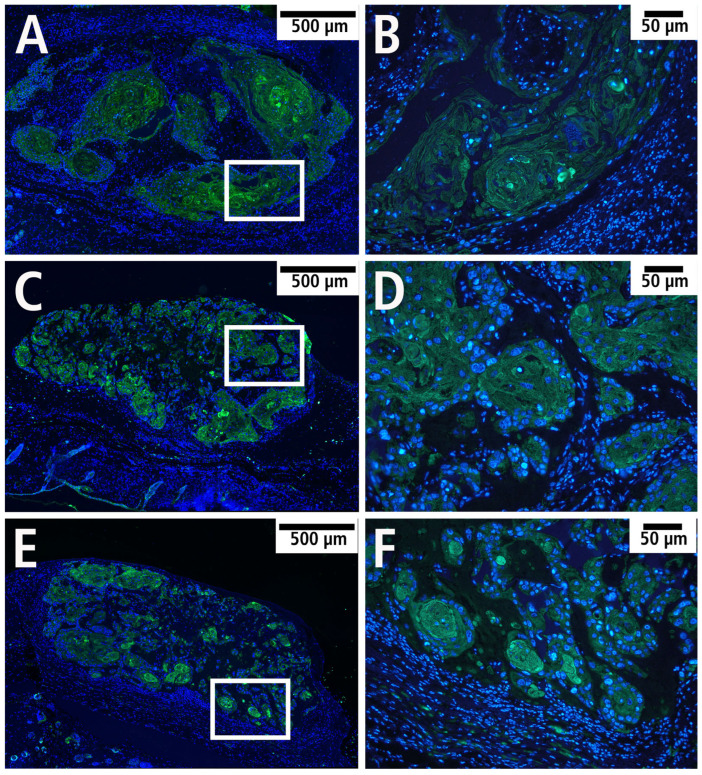
Epithelial carcinoma cells visualized by immunofluorescence with labeling of cytokeratin (green) and cell nuclei (blue) in the PDS^®^ group (**A**,**B**), Preclude^®^ group (**C**,**D**), and Dualmesh^®^ group (**E**,**F**). (**B**,**D**,**F**): Magnification of the marked area (black rectangle) of the histological sections from (**A**,**C**,**E**).

**Figure 10:**
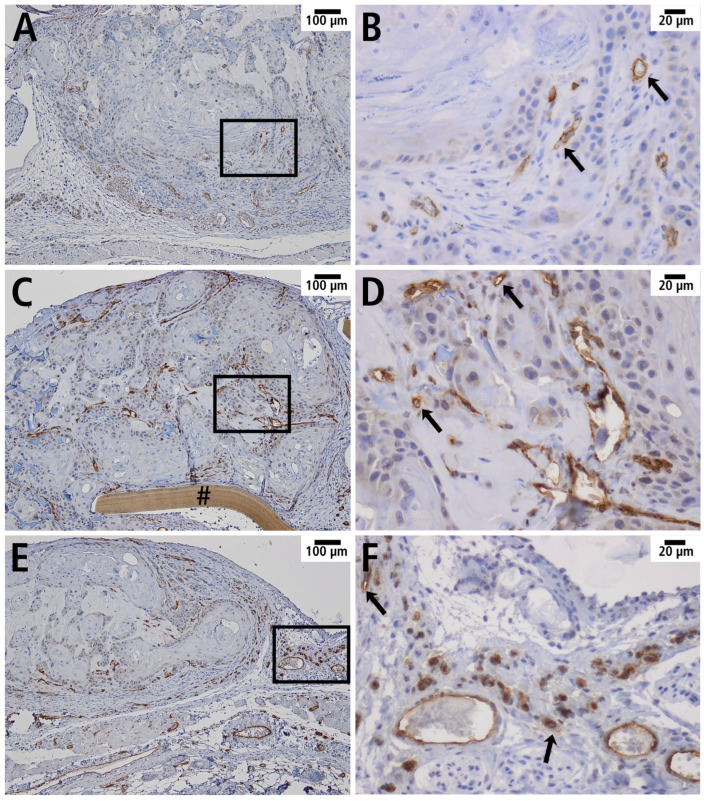
Manifest tumor with immunohistochemically labeled CD31 in overview (**A**,**C**,**E**) and in detail (**B**,**D**,**F**) in the PDS^®^ group (**A**,**B**), Preclude^®^ group (**C**,**D**), and Dualmesh^®^ group (**E**,**F**). Vascular endothelium of tumor vessels is marked with arrows. (**B**,**D**,**F**): Magnification of the marked area (black rectangle) of the histological sections from (**A**,**C**,**E**). # Remnant of the Preclude^®^ membrane.

Furthermore, squamous cell carcinoma cells were visualized using immunofluorescence by labeling cytokeratin to identify epithelial cells (Figure 9). Capillaries were visualized by immunohistochemical staining for CD31 to identify endothelial cells. CD31 was successfully detected in all groups, with the endothelial cells localized mainly in the marginal area of the tumor (Figure 10).

## 4. Discussion

The prognosis for patients with oral squamous cell carcinoma is still poor and novel therapeutic approaches are necessary. Therefore, a fast and safe method for preclinical testing of new treatments would be very helpful in accelerating therapeutic development.

So far, no animal model has made it possible to evaluate how effective new anti-angiogenic drugs are in treating advanced tumors. Therefore, a model that combines the advantages of intravital fluorescence microscopy with those of a solid tumor model is highly desirable. To enable observation and analysis of a tumor without any restrictions, a tumor must be positioned centrally within the dorsal skinfold chamber. Furthermore, adequate vascularization and the blood supply to the tumor mass must be guaranteed, even after preparation of the skinfold chamber. This requirement can only be achieved if the tumor grows along the dorsal skin, rather than invading the underlying muscle tissue. To ensure this, it is essential that the tumor remains separated from the musculature, preventing its attachment and deep tissue infiltration. Previous studies have already demonstrated the feasibility of reliably inducing squamous cell carcinoma through subcutaneous injection of tumor cells [12], providing a foundation for the development of such a model.

However, controlling the precise localization of the induced tumor remains a challenge in subcutaneous tumor models. To address this, we employed a carrier material in which tumor cells were embedded before the implantation, combined with a surgical membrane, which directed tumor expansion toward a defined anatomical location. The primary selection criterion for the membranes used was biological compatibility. All three materials tested—Dualmesh^®^, Preclude^®^, PDS^®^ plate—have previously demonstrated reliable biocompatibility in various clinical settings, including abdominal (Dualmesh^®^), cardiovascular (Preclude^®^), and oral, maxillofacial, and facial surgeries (PDS^®^ plate) [23,24,25].

Another important requirement was minimal adhesion between the tumor mass and the separating membrane. As discussed above, the different membranes were essential for guided tumor growth. They guaranteed that the vascular supply of the tumor only originated from the dorsal skin and the dorsal cutaneous muscle and prevented vascularization originating from the superficial muscles of the body. This specific and directed vascularization of the tumor was the essential prerequisite for the success of the later preparation of the skinfold chamber with a vascularized tumor inside. Without the use of the different membranes, the vascularization of the tumor would originate from the dorsal skin and the superficial muscles of the body. In such a case, the preparation of the skinfold chamber with the tumor inside would only be possible if the vessels, which originate from the superficial muscles of the body are disrupted, which would result in a disturbed blood supply of the manifested tumor.

The use of different membranes represents a double-edged sword. While they are essential for directing the vascular supply of the tumor, they pose a challenge during the subsequent preparation of the dorsal skinfold chamber. If the membranes were to remain on the established tumor during chamber preparation, subsequent intravital microscopic analysis would no longer be feasible. It was therefore crucial that the membranes could be removed during preparation without causing damage to either the tumor itself or its supplying vascular system. Among the tested membranes, PDS^®^ plate showed superior performance. During preparation of the dorsal skinfold chamber, it allowed for easier and cleaner tumor separation, which represents a significant procedural advantage over the other materials evaluated.

Considering the spatial constraints of the dorsal skinfold chamber, a tumor diameter of 3–4 mm was optimal for this study. At this size, tumors require an independent vascular supply, as simple diffusion becomes insufficient for oxygen and nutrient delivery beyond a volume of approximately 1 mm^3^ [26]. Nonetheless, tumors of this dimension remain sufficiently small to be accommodated within the 12 mm observation window of the chamber, ensuring complete imaging coverage. The maximum tumor size feasible within the dorsal skinfold chamber represents only a relatively small tumor. This limitation is inherent to the model and to the dimensions of the chamber in the mouse. While larger tumors would certainly be desirable from a clinical perspective, our in vivo model is designed to enable direct observation of the vascular system of established tumors by intravital microscopy, which necessarily requires certain constraints.

The model is intended for the investigation of anti-angiogenic agents. In contrast to existing approaches that only allow indirect assessment (e.g., tumor regression) [27,28], our model enables direct analysis of the tumor vasculature. This represents one specific aspect among many therapeutic approaches, but no single in vivo model can comprehensively address all pharmacological options. Complementary models with their respective strengths and limitations will always be required.

During tumor induction, it is important to handle the cell carrier carefully to prevent inadvertent release of tumor cells, which can lead to tumor growth outside the intended location and increase the risk of hematogenous or lymphatic metastasis. Cao et al. [29] described lymphatic metastasis after tumor induction with SCC-15 cells in an immunocompromised mouse model (NOD.Cg mouse) within only five weeks. These findings underscore the necessity of rigorous monitoring of experimental animals, especially during the tumor induction phase, to promptly identify and manage potential metastatic spread.

Compared to conventional histology, intravital fluorescence microscopy allows the direct visualization of changes in the vasculature that supplies the growing tumor for a certain period. These changes are the result of the interaction between tumor cells and the surrounding host tissue in living animals. Although the contributing factors cannot be visualized directly, either positive or negative can be quantified by microvascular parameters like functional capillary density or leukocyte–endothelial cell interactions (when inflammatory reactions are to be considered). In the present study, we reduced the duration of microvascular observation from approximately 14 days to 6 days. This was sufficient to assess whether the tumor remained adequately vascularized following the preparation of the dorsal skinfold chamber.

We showed that the shortened observation period was sufficient, as no regression of microcirculation was found in any of the animals after the preparation of the dorsal skinfold chamber. This demonstrated the successful establishment of a reliable murine model for intravital microscopic examination of manifested tumors within the dorsal skinfold chamber. This result is supported by a significant increase in functional capillary density in the tumor areas during the observation period.

Previous studies have shown that the implantation of synthetic as well as biological materials into a dorsal skinfold chamber can induce neoangiogenesis [30,31]. In the present study, we confirmed the angiogenic responses in subcutaneously implanted cells that had already formed a manifest tumor. The tumor was allowed to grow for up to six weeks prior to chamber implantation, providing sufficient time for substantial vascular development and tumor progression to occur. Therefore, the formation of new blood vessels was observed in all experimental groups, which demonstrated a tumor-induced angiogenesis, potentially driven by the secretion of pro-angiogenic factors such as vascular endothelial growth factor [32].

However, during the surgical preparation of the dorsal skinfold chamber, not all vessels supplying the tumor can be preserved, especially when tumors reach a diameter of more than 4 mm [26]. As a consequence, this leads to local hypoxia, apoptosis [33,34], and necrosis, which lead to molecular signaling cascades that further stimulate endothelial cell proliferation and promote compensatory angiogenesis in peritumoral regions [35].

Polydioxanone (PDS^®^) is a synthetic, resorbable polymer degradable by hydrolysis in vivo and has been extensively studied as a suture material due to its biocompatibility and well-characterized foreign body response [36,37]. Such inflammatory reactions are known to promote angiogenesis through leukocyte activation and the release of pro-angiogenic mediators [38]. This fact is a possible explanation for the high functional capillary density using the PDS^®^ plate. Therefore, it could be that molecules of the PDS^®^ plate induce local inflammatory response triggered by the degradation of the products, which further promote tumor angiogenesis.

The macromolecular leakage observed in this study was comparable to previous reports using dorsal skinfold chamber [39]. This is an important finding with regard to a potential extension of the observation period, as abundant macromolecular leakage and elevated vascular permeability can reduce the vessels’ optical contrast and hinder reliable intravital microscopy [40]. Based on our results, an extension of the examination period in future studies will not entail changes in the proposed procedure.

Furthermore, the model presented here can be adapted for use in other intravital microscopy settings. For example, the femur window or the calvaria bone chamber could enable investigation of angiogenesis in brain tumors or bone-metastasizing cancers and facilitate the preclinical evaluation of targeted therapies [41,42,43].

While various studies have previously employed tumor cells in intravital microscopy, our approach differs fundamentally in the methodology of tumor induction and observation. Most prior models involve the injection of tumor cells after chamber preparation or directly into the dorsal skin within the chamber. In such models, it remains uncertain whether the injected cells maintain normal biological behavior under these conditions [44,45,46].

In contrast, our study establishes a model based on the induction of pre-formed, manifest tumors before preparation of the skinfold chamber, enabling intravital imaging of established solid tumor tissue. This allows for more accurate observation of tumor progression, vascularization, and therapeutic response over time. Notably, models based on cell injection directly followed by an imaging procedure—such as those described by van Rheenen and colleagues—often permit only short-term imaging (up to 12 h) and are not suited for longitudinal studies [47,48,49].

## 5. Conclusions

The presented model enables, for the first time, intravital microscopy of pre-formed, well-vascularized solid tumors within the dorsal skinfold chamber over several days, allowing more realistic preclinical testing of novel therapies. By combining a cell carrier with a surgical membrane, tumor growth is directed along the skin rather than into muscle tissue, facilitating both imaging and tumor removal. Among the tested materials, PDS^®^ plate proved most suitable, enabling preparation of the skinfold chamber without disturbing the tumor vasculature. This model represents a substantial improvement over traditional cell injection approaches, which often limit imaging to short-term observations. Moreover, it lays the foundation for standardized, reproducible tumor models and opens the possibility for personalized in vivo testing of therapeutic responses.

## Figures and Tables

**Table 1:** Blood cell velocity and vessel diameter of tumor capillary venules.

	Day 0	Day 3	Day 6
Blood cell velocity [µm/s]			
PDS^®^	377.0 ± 63.4	446.9 ± 72.2	435.1 ± 74.7
Preclude^®^	346.4 ± 123.9	469.3 ± 58.1	486.6 ± 133.0
Dualmesh^®^	341.8 ± 15.3	465.5 ± 45.6	553.7 ± 128.1
Vessel diameter [µm]			
PDS^®^	31.4 ± 4.1	26.5 ± 4.2	31.5 ± 6.1
Preclude^®^	26.7 ± 3.5	33.3 ± 5.5	34.9 ± 2.6
Dualmesh^®^	28.0 ± 5.7	29.4 ± 3.1	32.3 ± 3.9

Blood cell velocity and vessel diameter of tumor capillary venules in PDS^®^, Preclude^®^, and Dualmesh^®^ groups immediately (day 0), as well as 3 and 6 days after fixing the dorsal skinfold chamber. Values represent means ± SEM.

**Table 2:** Microhemodynamics of tumor capillary venules.

	Day 0	Day 3	Day 6
Volumetric blood flow [pL/s]			
PDS^®^	189.4 ± 63.4	156.0 ± 53.5	208.3 ± 45.5
Preclude^®^	123.6 ± 64.4	* 258.4 ± 85.2	* 291.7 ± 81.7
Dualmesh^®^	134.0 ± 48.2	198.4 ± 41.5	^#^ 289.8 ± 117.6
Wall shear rate [s^−1^]			
PDS^®^	96.1 ± 0.9	137.8 ± 3.7	114.7 ± 4.1
Preclude^®^	105.5 ± 3.8	116.1 ± 2.7	112.3 ± 3.4
Dualmesh^®^	100.9 ± 2.5	127.7 ± 2.1	137.9 ± 3.3

Volumetric blood flow and wall shear rates of tumor capillary venules in PDS^®^, Preclude^®^, and Dualmesh^®^ groups immediately (day 0), as well as 3 and 6 days after fixing the dorsal skinfold chamber. Values represent means ± SEM. * *p* < 0.05 vs. Preclude^®^ and Dualmesh^®^ on day 0. ^#^ *p* < 0.05 vs. Preclude^®^ and Dualmesh^®^ on day 0.

## Data Availability

All data generated or analyzed during this study are included in this published article. The dataset is available on request from the authors.

## Abbreviations

The following abbreviations are used in this manuscript:

NSG	NOD scid gamma
FITC	fluorescein isothiocyanate
DAPI	4′,6-diamidino-2-phenylindole
SEM	standard error of mean
SD	standard deviation
ANOVA	analysis of variance
